# Policy space and pro-health equity national policymaking: a case study of Myanmar during political transition (2006–16)

**DOI:** 10.1093/heapol/czac076

**Published:** 2022-09-09

**Authors:** Fiona Campbell, Than Tun Sein, Thant Sin Htoo, Wai Yee Krystal Khine, Natasha Howard, Dina Balabanova

**Affiliations:** Department of Global Health and Development, London School of Hygiene and Tropical Medicine, 15-17 Tavistock Place, London WC1H 9SH, United Kingdom; Department of Medical Research, Formerly Ministry of Health, No. 5 Ziwaka Road, Yangon 11191, Myanmar; Ministry of Health and Sports, Minister’s Office, No. 249 Theinbyu Road, Mingalar Taung Nyunt Township, Yangon, Myanmar; Formerly 3MDG Fund, No. 12 O Pyi Thu Street, Yangon, Myanmar; Department of Global Health and Development, London School of Hygiene and Tropical Medicine, 15-17 Tavistock Place, London WC1H 9SH, United Kingdom; Saw Swee Hock School of Public Health, National University of Singapore and National University Health System, 12 Science Drive 2, Singapore 117549, Singapore; Department of Global Health and Development, London School of Hygiene and Tropical Medicine, 15-17 Tavistock Place, London WC1H 9SH, United Kingdom

**Keywords:** Policy space, health equity, sustainable development goals, policy analysis, political transition, policymaking

## Abstract

Health equity is central to achieving sustainable development goals and COVID-19 has emphasized its importance. Ensuring health equity is prominent in policy discussions and decision-making is a critical challenge in all countries. Understanding the policy space for actors to promote health equity in the policy process may help to strengthen prioritization of equity in policy and programme discussions and decisions. Authors developed a conceptual framework for policy space based on a narrative literature review. This comprised five key elements and their associated factors, i.e. context, policy circumstances, policy characteristics, actor engagement and policy spaces. Authors then applied it in Myanmar during a period of political transition, using a qualitative case study design. Findings showed that political transition provided an important ‘policy window’ to develop more equitable health policy in Myanmar. Changing policy circumstances offered opportunities for advancing pro-equity policy. However, lack of visibility of health equity and long-standing inequalities were important challenges to policy space. Within a changing context, actors at individual and organizational levels used a range of policy spaces to advance pro-equity health policy. Learning from using the framework in Myanmar was incorporated into a revised framework. Application of this revised framework could provide valuable insights into the opportunities to promote a pro-health equity approach across policy and programme discussions and decision-making for actors trying to promote equity in other transition and non-transition contexts.

Key messages

- We include key elements of policy space identified in the literature in a single framework.
- We tested policy space elements for individual and collective contributions to conceptualization and use of policy space to promote health equity in political transition.
- Stronger focus on policy space for health equity can help identify opportunities to prioritize and advance equity at different transition stages.

## Introduction

Health equity is at the core of the sustainable development goals (SDGs), specifically SDG 3, *Ensure healthy lives and promote well-being for all at all ages* (WHO, 2016) and many other international legal frameworks and guidance. Policymakers increasingly recognize the need to tackle health inequalities at country and global levels (Hosseinpoor *et al.*, 2015) and addressing these is seen as central to effective responses to COVID-19. However, gaps between intentions and reality in both policy and practice are significant (Friedman, 2015; 2019). Lack of attention to equity in policy discussions (Ridde, 2008) and poor articulation of health equity goals within policies (McIntyre and Gilson, 2002) have hampered efforts to address disparities in health outcomes and healthcare access. Using emerging opportunities to place health equity prominently on the policy agenda and designing policies to advance it are critical for strengthening health systems (Chopra, 2013) and achieving the SDGs (Marmot and Bell, 2018).

The extent to which actors successfully create and act on opportunities to promote health equity as a policy issue is dependent on multiple factors. These include how actors conceptualize equity, the interplay between individual and institutional actors and their context and the power and interest of different actors to shape equity-enhancing policies (Walt and Gilson, 1994; Shiffman and Smith, 2007; Tantivess and Walt, 2008; Sriram *et al.*, 2018).

One challenge is that the definition of *health equity* is not universally agreed (Braveman, 2006) and different meanings and conceptualizations have implications for the policy options put forward and ultimately adopted. Health equity is principally concerned with justice and fairness (Whitehead, 1992; Peter and Evans, 2001), while also being closely aligned with health rights (Braveman, 2010). It may be defined in terms of ‘health outcome’ such as *status or wellbeing* (Whitehead, 1992; Braveman and Gruskin, 2003); in terms of ‘*healthcare’* both as *access to* and *use of* (Mooney, 1983; Culver and Wagstaff, 1993) and as a broader concept incorporating multiple dimensions relating to social justice (Sen, 2002, p. 660). The present study used Braveman and Gruskin’s (2003, p. 254) clear definition of health equity: ‘*the absence of systematic disparities in health (or [their] social determinants) between social groups who have different levels of underlying social advantage/disadvantage*’ as its starting point, further exploring its meaning during research.

The concept of *policy space,* which is also central to this study, is commonly used to describe opportunities to intervene in policy processes (Sutton, 1999). A body of literature developed dimensions of the term and described its use (Koivusalo *et al.*, 2009). The term denotes the ‘*room*’ actors—both governmental and non-governmental—have to address a policy issue (Sutton, 1999). This includes the room available to government actors to develop policies (Koivusalo *et al.*, 2009) and room for non-governmental actors, including civil society, to engage with the policy process (Crichton, 2008).

Policy space often involves the existence of multiple actors collaborating or negotiating from different positions. While government actors often drive the policy process, non-state actors can play a critical role in shaping policy dialogue and the ultimate outcome. Therefore, the policy space available to actors is shaped by opportunities to participate in and influence these policy processes (Crichton, 2008). This is facilitated through various formal and informal structures, mechanisms and networks (Walt and Gilson, 1994; Lewis, 2006; Shiffman and Smith, 2007; Tantivess and Walt, 2008).

Few studies have explored the use of policy space as a framework to support policy analysis (Crichton, 2008). These have examined the space that a particular actor (often governmental) has in developing policy (ibid) or the space available to multiple actors around a specific issue such as family planning (ibid). This study aimed to examine how the concept of *policy space* can be applied in identifying and promoting opportunities to prioritize equity in policies and programmes. Building on previous scholarship on the concept, this study proposes a conceptual framework that can be used to identify health equity policy space in a dynamic context of sociopolitical change. A range of social and economic factors, including poverty, conflict, ethnicity, status and geographical isolation, were seen to increase vulnerability to worsened health outcomes in Myanmar (World Bank, 2014), providing a useful context in which to examine the application of this framework in promoting a pro-health equity approach within the policy process during a decade of sociopolitical transition in Myanmar (2006–16) and provide lessons for engagement in future policy processes.

## Methodology

### Study design and research question

We chose a case study design set in a historical context underpinned by a conceptual framework for policy space described below. We examined the question of how policy space has enabled or obstructed progress towards health equity in Myanmar during 2006–16 and key factors influencing this process.

### Framework development

We developed a conceptual framework to assess the policy space for health equity in Myanmar during a particular time-period. We derived this framework based on critically reviewing and adapting existing frameworks following the steps outlined by Maxwell (2013, chapter 3). We conducted a narrative literature review to identify existing definitions and uses of the term ‘health equity’ and conceptualizations and research on ‘policy space’. The review first examined the multiple definitions of health equity and its links with ethical and human rights principles (Braveman, 2006). We then identified three main frameworks with direct relevance to policy space. First, Grindle and Thomas (1991) framework outlines ‘room for manoeuvre’ for government policymakers and identifies three key factors influencing this space: (1) ‘environmental context’, encompassing policymakers’ views and external domestic and international influences, (2) ‘agenda-setting circumstances’, relating to policymaking environment and its influence on decision-making and (3) ‘policy characteristics’, concerned with how a policy may affect the decision-making environment. Second, Crichton’s (2008) framework builds on Grindle and Thomas’ work to examine the space for a health issue from multiple stakeholder viewpoints, i.e. beyond governmental actor perspectives. Third, McGee (2004), Gaventa (2006) and Brock *et al*. (2001) examine ‘policy spaces’ available to a range of actors to influence the policy process (Gaventa, 2004; 2006; McGee, 2004). We also reviewed research on globalization, trade and national policy space (Koivusalo *et al.*, 2009; Milner, 2009) and several health equity frameworks (Bornemisza *et al.*, 2010; Gopalan *et al.*, 2011) for additional insights into concepts of policy space, health equity and their potential interaction. Finally, we examined policy frameworks with elements relevant to policy space in the policy analysis literature, including Kingdon (2011), Walt and Gilson (1994) and Shiffman and Smith (2007) to examine the role of different elements and characteristics of policy space.

Our analysis identified five key elements and associated factors (Table 1) that provided the initial framework to explore the complex interplay between factors in the Myanmar context, building on the experience and knowledge of authors and study participants and allowing new insights to emerge inductively as part of the research process to shape later versions of the framework.

**Table 1.** Conceptual framework elements and factors for health equity policy space (based on the literature)

Framework element	Relation to policy space (for health equity)	Factors identified as influencing policy space (with reference to health equity)
Contextual factors	Relates to factors that are *“pre-existing”* and affect how decision makers view the problem (Crichton, 2008) of health equity	Historical, social, cultural, economic factors (Crichton, 2008; Grindle and Thomas, 1991) Situational factors, e.g. a humanitarian crisis, political transition (Crichton, 2008; Shiffman and Smith, 2007) “Policy windows” (Shiffman and Smith, 2007; Kingdon, 2011) International context such as agreements, treaties, loans, development assistance (Koivusalo *et al.*, 2009; Koivusalo, 2014); global governance structure (Shiffman and Smith, 2007) Views/opinions held on health equity (Grindle and Thomas, 1991; McGee, 2004).
Policy circumstances	Relates to factors affecting the *“dynamics of the decision-making”* process (Crichton, 2008) on health equity	Pressure/urgency for reform or action on issue (Grindle and Thomas, 1991; Crichton, 2008) Priority given to issue (Walt and Gilson, 1994) “Crisis situation” or “politics as usual” (Grindle and Thomas, 1991; Crichton, 2008) Available strategies to address issue (Crichton, 2008) Policy-making process (Gopalan, 2011)
Policy characteristics	Relates to factors that impact on *“acceptability*” by decision makers (Crichton, 2008) of health equity	Visibility of issue (Crichton, 2008) How different sections of population affected by issue and efforts to address (Crichton, 2008) Evidence on problem (scale etc) of health inequities (Shiffman and Smith, 2007) Resources/efforts needed to address issue (Grindle and Thomas, 1991; Crichton, 2008; Shiffman and Smith, 2007; Gopalan *et al.*, 2011)
Actor engagement	Relates to how actors engage with and use policy space to advance (or otherwise) the agenda (Crichton, 2008) on health equity	Range of actors present/degree of involvement (Gopalan, 2011; Brock *et al.*, 2001) Power/influence of different actors (Gopalan *et al.*, 2011; Shiffman and Smith, 2007; Brock *et al.*, 2001) Actor interaction (Ir *et al.*, 2010) Capacity of actors to engage in policy (Gopalan, 2011)
Policy spaces	Relates to the nature and types of spaces available to engage on the issue (Gaventa, 2006) of health equity	The nature of different spaces, e.g. “closed”, “invited”, “Claimed/created”, “visible”, “invisible” (Brock *et al.*, 2001; Gaventa, 2006), “conceptual”, “bureaucratic”, “practical” (IDS, 2006) “Rules of engagement” (formal and informal) (Gaventa, 2004) Inclusion of different actors in spaces (McGee, 2004; Brock *et al.*, 2001) Creation of spaces (McGee, 2004; Brock *et al.*, 2001) Governance of spaces (McGee, 2004) How spaces are used for decisions on funding and coverage of programmes (Bornemisza *et al.*, 2010; Buse and Walt, 1997)

**Figure 1.**
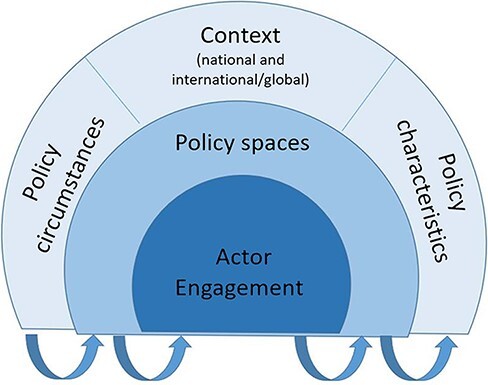
Diagrammatic representation of policy space framework for health equity (colour preferred but not essential) (based on key elements from Grindle and Thomas, 1991; Brock *et al.*, 2001; McGee, 2004; Crichton, 2008; Gaventa, 2004 and others)

Figure 1 shows these elements diagrammatically. In the outer arc, the three elements of *context, policy circumstances* and *policy characteristics* shape the wider environment for health equity policy. In the middle arc, *policy spaces* present opportunities and challenges for engagement on health equity. At the core, *actors* engage with policy spaces and the wider environment to promote or obstruct the health equity agenda. These elements should be viewed as constantly interacting and influencing each other across the different domains.

### Study setting

During the study period, Myanmar underwent a sociopolitical transition from a decades-long period of authoritarian rule towards democracy. This transition occurred in several stages. First, the period before 2010, under a military government and the State Peace and Development Council led by Senior General Than Shwe. Second, a period starting from late 2010 to early 2011 and the election of the Union Solidarity and Development Party (USDP), overseen by a new ‘quasi-civilian’ government under President Thein Sein. Third, the period from late 2015 to early 2016 and election of a new National League for Democracy-led government under President Htin Kyaw, with Daw Aung San Suu Kyi as State Counsellor. The decade under study thus contained two successive periods of political transition, together with a stage that in hindsight can be seen as pre-transition. With successive new governments, engagement between government and a range of health and other actors intensified (IHP, 2013; Risso-Gill *et al.*, 2014). The context and period chosen thus offered a unique opportunity to study a dynamic process of change in Myanmar, with an opportunity to examine changes in policy space over time. Since this study was undertaken, Myanmar military efforts from early 2021 to reassert control following the 2020 election and suppression of subsequent civilian resistance have derailed the transition process (Price, 2021) with implications for health equity policy space.

### Data collection

We employed two data collection approaches. The first one recognized ‘*people as informants*’ and obtained their assessments and views as immersed participants through semi-structured interviews. The second focused on the ‘*documentation*’ of key policy and other decision-making processes and programmes in Myanmar relevant to health equity in 2006–16 (Potter and Subrahmanian, 1998).

Data collection took place principally in Myanmar by the first author and Myanmar co-authors. We purposively recruited actors involved in key health policy discussion and policymaking fora over the 10-year period. We identified for interview actors holding key positions within national or international agencies and membership of at least one key forum during the decade of interest. Invitations were sent to ensure a total of 25–30 interviewees across actor groups, assuming a level of non-response. Table 2 describes the characteristics of the 29 interviewees, of 31 invited by email, who responded positively. In total, 13 were women and 16 men, 14 were Myanmar and 15 international, across the range of actor groups, including representatives of government departments, bilateral donors, United Nations (UN) and national and international NGOs. The first author conducted interviews in English using a question guide informed by the literature and expert opinion. Interviews were audio-recorded in all but two cases and transcribed by the first author, with anonymity and confidentiality maintained throughout by using identification codes and securely storing transcripts.

**Table 2.** Key policy space factors illuminated by qualitative research in Myanmar

Key element	Key factors illuminated by use of framework in Myanmar
Context	Historical influences e.g. legacy of military regime; application of sanctions by some governments International/global influences e.g. sanctions imposed by some governments e.g. USA and UK Political factors e.g. elections in 2011 and 2015, National Ceasefire Agreement (NCA) Social factors e.g. range of languages, health system challenges, trust in government services Legal factors e.g. change in laws/articles Key events e.g. Cyclone Nargis in 2008; Views on health equity held by different actors
Policy circumstances	Low prioritization of health equity in pre-transition period Political imperative to address health equity at different times e.g. pre- and post- election pledges Changing nature of policy making process during the political transition e.g. move from exclusive top-down approach to more inclusive approach to policy making over decade
Policy characteristics	Lack of visibility of health inequalities/inequity Challenge of long-standing inequities e.g. consideration of health equity goal; practical and administrative challenges; balancing with “do no harm”; linking with peace process; resources needed to address the issue
Actor engagement	Actor availability for engagement in health policy processes e.g. mix and increase of actors over time Scope of actor engagement in policy processes Actor agency (individual, organizational) Programmes to address health inequalities e.g. targeting MDGs, expanding coverage of services, resource allocation to marginalized groups and areas of country Actions to expand actor engagement in policy discussions Actions to expand visibility of health inequalities/inequity
Policy spaces	Types of space – Conceptual space e.g. UHC as way of conceptualizing health equity – Formal/-Informal spaces e.g. programme and policy fora such as CCM, MHSCC, NHC/ “tea break advocacy” – Exclusive/Inclusive spaces e.g. national policy fora e.g. NHC/NHP – Key events e.g. Elections, NCA, Cyclone Nargis – Financial space e.g. Increasing government budget post 2011; financing from key actors such as Global Fund, World Bank – Programme space e.g. various programmes to support health – Historical e.g. legacy of sanctions, legacy of military regime – Visibility space e.g. availability and use of data on inequalities Use of space – For promoting discussion on health equity e.g. promoting ideas on health equity – For increasing coverage of programmes e.g. 3DF – For promoting visibility of health equity e.g. Gavi-HSS, PONREPP and use of hard to reach terminology – For accountability e.g. increase in media attention on health

A range of key policy and programme documents with a bearing on health equity were identified and reviewed for information on key aspects of health equity policy space. These included policy documents relating to national and international health and other processes, such as national health plans and policies and key programme and other reports. In addition, all documents mentioned by the interviewees were traced and included. Selected documents were reviewed against a document monitoring sheet that aimed to capture information relevant to policy space for health equity and actor engagement, following the methodology outlined by Prior (2011).

### Analysis

We analysed transcript and document data using thematic analysis (Saldaña, 2016). The coding process was used to identify key events and actions to forward health equity and identify constraints as outlined by Gibbs (2007). Initial codes were generated and used to code all transcripts. A final list of codes was assembled into a hierarchy around key themes. Key themes were then used to develop a series of extraction tables, populated with information from interviews and documents and used to critically interrogate the value of framework elements and factors and provide insights into the policy space available for health equity in Myanmar over the decade under consideration.

## Findings

Findings are presented against elements of policy space for health equity in Myanmar, as identified in the conceptual framework, throughout the decade under review.

### Contextual factors

Actors consistently highlighted contextual factors as key to the policy space for health equity in Myanmar. Two important aspects were identified for this domain; first, those impacting the *context of policy space* (Grindle and Thomas, 1991, p. 184) and second, the views of actors on health equity. In the first category, many interviews highlighted historical factors, such as the legacy of a military government and international sanctions by governments, including the USA and UK, as essential factors that shaped the overall environment for health policy and health equity. Historical factors were described as limiting the space for health equity in multiple ways in the early part of the decade, including by reducing financial space and limiting opportunities to discuss the challenge of inequities.

The international political context and the sanctions … affected the type of aid that came, and the volume of aid, so […] a fairly low volume of aid (INT7-2)

Later, key events, such as Cyclone Nargis in 2008, presented opportunities to change the discourse and nature of engagement by introducing new international actors and encouraging the growth and participation of national civil society. After 2010, political events in 2010/11 and in 2015/16 were identified as key to providing a critical ‘policy window’ for health equity and opening policy space. For example, most participants highlighted the 2010 elections and new government in early 2011 as contributing to subsequent easing and eventual lifting of sanctions by international actors in 2012. This was an important turning point and an opportunity to advance health and health equity. Key influences identified included increased funding for health from government and international sources and new opportunities for engagement between the Ministry of Health (subsequently Ministry of Health and Sports) and a range of health actors, including ethnic health organizations, and new opportunities to discuss challenges within the health system by a broader set of actors. These developments were then built upon following subsequent political events from 2015/16. Table 3 illustrates key shifts with a selective overview of political periods and corresponding health policies. Another important historical influence recognized in interviews was the legacy of conflict, which has affected many parts of the country over many decades, negatively affecting the opportunities for provision of social services and development of health equity (WHO, 2022).

**Table 3.** Summary overview of development of health policies and plans prior to and during study period (summarized from Health in Transition, 2014 and National Health Plans 2006–2011; 2011–2016; 2016–2021)

Period	Key political period	Key health policy
Pre--2006	1988–1992 -- SLORC -- military 1992 SDPC -- military	National Health Policy 1993 Health financing reforms (1990s) Development of drug revolving Fund for essential drugs Introduction of User charges Private service in public hospitals Other policies: Establishment of health care facilities in border areas
2006–2011	SDPC -- military	National Health Plan 2006–2011 PHC approach with emphasis on equity, preventive services, community involvement, multisectoral approach
2011–2016	USDP -- “quasi-civilian”	National Health Plan 2011–2016 PHC at the centre of UHC Improving equity Improving efficiency in Strengthening capacities for UHC Key policies: Hospital Equity Fund MCH Voucher scheme Township based health protection scheme
2016–2021	NLD-led government	National Health Plan 2016–2021 Emphasis on PHC delivered at township level and below Focus on access to essential health services for population in phased manner Considers health care providers outside the MoHS

The views of actors also influenced the discussion on health equity. Most actors described health equity in relation to measures of ‘access’ to health services.

[Health equity is…] health services provided to people depending on their health status as well as their wealth status (INT27-1)

health equity to me would be about access equity, it doesn’t mean we are all going to get the same service, but it means that everyone in society is able to access the same service (INT8-2)

Several respondents linked health equity explicitly to Universal Health Coverage (UHC) or described the national strategy for UHC, particularly the delivery of an essential health package, as an opportunity to discuss and promote a pro-equity agenda.

For health equity, I usually refer to the universal health coverage concept, but I will not say universal health coverage heading, but I mean the concept is universal health coverage (INT14-1)

A few respondents framed health equity from the perspective of resource allocation, noting the appreciable inequality in current allocations, including in relation to the distribution of human resources across the country.

Finally, a minority referenced health equity in terms of health outcomes.

We see health inequity as health[y] life expectancy (INT18-1)

### Policy circumstances

Actors highlighted the importance of policy circumstances in determining the space for health equity, identifying key elements in a changing political context that were critical for enhancing space for health equity in Myanmar. Both government and non-governmental actors described the increased political imperative to prioritize specific social issues and address inequalities following the 2010 and 2015 elections, as key to raising the profile of health equity on the political agenda. This was reflected in the changing nature of the policy process over this time, with increased opportunities to discuss health equity, identified in many interviews as an important factor in expanding policy space. For example, before 2010, only a limited number of actors could access formal policy discussions, i.e. those within government policy processes and a few trusted UN agencies. Outside these government processes, national and international actors found ways to shift the policy agenda in a pro-equity direction, albeit incrementally, using a variety of approaches. These included promoting ideas and discussion on equity using specific terminology such as ‘hard-to-reach’ in programme discussions and designing specific programmes with a clear equity focus, such as the post-Nargis Recovery and Preparedness Plan (PONREPP) after Cyclone Nargis, which used a township planning approach originally applied by the Gavi-HSS programme and helped to examine health equity in service provision and use, as well as framing of programmes aimed at supporting pro-health equity initiatives (i.e. including a pilot programme on the use of vouchers and hospital equity funds). Discussions around three diseases (i.e. HIV, TB, malaria) within the Global Fund Country Coordination Mechanism (CCM) and other technical strategy groups also enabled discussion on health equity from a disease-specific perspective. These programmatic fora played a critical role in expanding opportunities for policy engagement and discussion for a wider set of national and international actors with the Ministry of Health (MoH).

I think definitely the […] country coordination mechanisms […] did result in a forum where you could talk about inequities. I mean, they were inequities as they related to the way in which, well either the way in which inequities resulted in disease burden for TB, HIV and malaria or the converse, […] the way in which the diseases themselves impacted on different populations in different ways (INT4-2)

From 2011, the expanded political space was accompanied by a gradual opening-up of the policy process for discussion and action on health equity. Several actors saw this as exemplified in the increased opportunities to discuss health sector challenges within formal spaces, e.g. the formation of the Myanmar Health Sector Coordination Committee (MHSCC) with a broader range of health stakeholders. From 2015, further expansion of the policy space was exemplified by a new process adopted for National Health Plan (2017–21) development. This process presented a significant step forward in supporting wider discussion of equity and allocation of supportive resources and recognizing a range of actors (e.g. policymakers from the private sector, Ethnic Health Organization [EHO] representatives) who worked with Ministry and UN actors to develop the new plan aiming for universal health coverage and a specific equity focus.

2011–2012 is for the international opening up […], but 2015–2016 is opening up of ethnic health organisations, ethnic groups, armed groups and then the CBOs, never ever in the Ministry of Health’s history did they ever recognise the work of NGOs and CBOs (INT19-1)

These shifts were reflected in policy documents from this period, including the 2013 ‘Strategic Directions for UHC’ presented by MoH, the 2016 ‘Programme of Health Reforms’ developed by the National Health Network, and the 2017–21 NHP from the Ministry of Health and Sports (MoHS), forwarding the goal of UHC. Increased government and international actor funding for health and other social sectors from 2011–12 also helped translate these policy commitments into concrete actions.

### Policy characteristics

Actors described a range of characteristics of policy development as having an important role in the policy space for health equity over the decade. A factor identified by many national and international actors was the increased visibility of health inequalities, linked to the availability and use of data on the depth and spread of health inequalities, creating space for more meaningful and action-oriented engagement on the issue. For example, pre-2011, limited availability of national data generally, and on health inequalities specifically, restricted opportunities to champion health equity or prioritize it on the policy agenda. In the absence of national data, programmatic data were critical in providing insights into the nature and scale of inequalities in healthcare access and health outcomes. However, data were severely limited in scope and restricted in dissemination due to political sensitivities. Further, availability of unreliable national health information hampered discussion on health inequalities. Some UN agencies appeared slow to request better data, while their endorsement of reports that potentially under-represented the scale of health challenges further worked against promoting the issue of health equity within the policy process.

From 2014, the availability of census data (2014) and Demographic and Health Survey (DHS) data (2015) enabled reflection on health inequalities across the country. However, limitations with these datasets continued to undermine opportunities to highlight inequalities and thus motivate action policies to promote health equity. This included a lack of disaggregation to levels that allowed identifying and discussing inequalities within states and regions and within and between different population groups. Additionally, concerns were raised at the time about the process and coverage of the Census and its political dimensions, including in relation to the identification of certain groups (ICG, 2014). Similarly, data from large donor-supported health programmes in the country provided limited information on their impact across population groups. These data challenges continued to constrain the policy space for health equity in Myanmar.

I don’t think there were any reasonably reliable statistics […] I think that the statistics that we do, the surveys that were supported by UN agencies, I don’t trust any of the data from those [days]. No, I think the first reliable data on health we are going to have is the DHS (INT9-2)

Interviews highlighted other critical factors that challenged efforts to address health equity in the post-2015 period. These included the political sensitivities of expanding the coverage of health services in areas not under government control. Further, as donor priorities changed with the shifting political situation and additional support to government was provided, it became difficult to provide finances directly to areas that were not under government control, including areas under EHOs.

### Actor engagement

Factors relating to context, policy circumstances and policy characteristics led to a range of ‘policy spaces’ offering both opportunities and challenges for actors to engage with the issue of health equity. The interviews highlighted the wide scope for actor engagement and use of different spaces to work on health equity, at the individual and organizational level, and across national and international groups. Respondents highlighted how the ability of actors to recognize and utilize the space for health equity varied by individual and group and over time. Individuals and organizations clearly identified and used space in various ways within the confines of their particular time and environment. For example, certain individuals in national and international agencies were described as seizing available opportunities to ‘do something different’. These opportunities often coalesced around identified ‘policy windows’ (noted above), such as the response to Cyclone Nargis or the re-entry of organizations into the country, e.g. the World Bank in 2012.

Interview respondents identified several national and international actors from the Ministry of Health (subsequently Ministry of Health and Sports), national agencies and international agencies, who clearly promoted a pro-equity agenda and placed health equity at the centre of their actions. This manifested in their use of language, encouragement of discussion on the issue and adoption of approaches to address it, whether targeted to specific groups or areas or through a holistic approach.

Individuals within institutions who, if you like, […] were working at the right level where they could take decisions and [ ] had […] a mandate within those institutions (INT15-2)

At an organizational level, programmes including PONREPP, Gavi-HSS and later the 3MDG Fund, used terminology such as ‘hard-to-reach’ and promoted a township planning approach, helping raise the visibility of health equity. Programmes addressing tuberculosis, malaria and HIV helped target these high-burden diseases within the context. However, in the view of some actors, their focus on selected diseases also fragmented the health improvement agenda and undermined their pro-equity credentials and national ownership of the health equity agenda. The shift of focus towards UHC and addressing health system issues later in the decade was seen as a more holistic approach to equity by both national and international actors, building on previous efforts to promote conceptual space through the use of ‘hard-to-reach’ terminology, targeting of the most vulnerable, and use of the township health planning approach.

Many respondents highlighted the influence of historical policy space on the present-day space with historical space as both a facilitator and challenge to current space. For example, where actors had identified and cultivated space to discuss health equity or developed programmes with a pro-equity focus in the pre-2011 or initial transition periods, these efforts then provided a modest but tangible base from which to expand space for health equity in the later period.

### Policy spaces

The respondents (representing the key actors) identified a range of policy spaces as important opportunities to further the health equity agenda over the period under consideration. These included conceptual, financial and programme spaces that allowed actors to address health equity in different ways, including the creation of important formal spaces that incorporated a wider range of actors in policy discussions. Examples of the latter involved fora connected to specific programmes, such as the Global Fund’s CCM offering an opportunity for civil society to engage in policy discussions and issue-specific groups and wider policy fora such as the MHSCC. Informal spaces also played an important role in advancing the discussion on health equity by both national and international actors. These included discussions around the margins of formal spaces or ‘under the radar’ programming, particularly before 2011.

There is sort of what I will call ‘sneaky spaces’. You know you can’t have health insurance, but what about a voucher scheme? That is not really health insurance is it? No, we don’t want to call it health insurance, and what about [a] hospital equity fund? That is not really health insurance, it is kind of health insurance but not really (INT7-2)

One important space identified in many interviews was the conceptual space provided by discussion on UHC that materialized towards the end of the research period, in which equity was central.

### Inter-connectedness of elements

While understanding the role of each individual element, the research indicated the interconnectedness of the framework elements and policy space for health equity. For example, the link between actors and other elements of policy space, such as context and policy circumstances, was evident in interviews (e.g. the shifts in policy space and openings in discussion and actions on health equity following cyclone Nargis and the arrival of new NGOs as well as the return of the World Bank). The research also highlighted how actors used the increasing space within the policy process presented by the changing political context and by global discussions such as UHC to advance ideas and initiatives related to health equity.

## Discussion

We employed the concept of policy space as an analytical tool to trace opportunities for addressing health equity in a transition context, applying our framework to Myanmar. Elements included in our framework, i.e. *context, policy circumstances, policy characteristics, actor engagement* and *policy spaces*, individually and collectively, helped determine the policy space for health equity in Myanmar in the study period. We found a range of factors influencing the availability of policy space for health equity in the country, shaped by national and international actors within a historical and changing political context, policy circumstances and policy characteristics. These factors created opportunities and challenges for engaging with health equity in various policy spaces. The emergence of policy spaces was important while unpacking how actors used these spaces was clearly critical to understanding the overall health equity policy space.

The analytical framework developed in this paper proved useful in identifying factors that need to be in place to increase space for health equity. These factors, outlined in Table 4, cluster around several lessons including: (1) political transition as an important ‘policy window’ for development of health equity policy; (2) definitions of health equity can help shape the scope of policy options; (3) changing policy circumstances present important opportunities as well as challenges for the policy space for health equity; (4) lack of visibility of inequalities reduces policy space for the issue; (5) longstanding health inequalities challenge the policy space for health equity; (6) pro-equity approaches can be adapted to each stage of transition and (7) actor engagement in policy space for health equity needs to be inclusive. Many of these factors have been recognized previously, such as the identification and use of ‘policy windows’ to further policy (Balabanova *et al.*, 2013), and several well-known frameworks exist for analysing the policy process, including Walt and Gilson’s ‘policy triangle’ (Walt and Gilson, 1994), Kingdon’s ‘multiple streams’ (Kingdon, 2011), and Shiffman and Smith’s ‘generation of political priority’ frameworks (Shiffman and Smith, 2007). However, while building on these seminal works, we argue that an expanded range of elements and factors are critical for effective policy space and should be considered in analyses.

**Table 4.** Revised framework for health equity policy space

Key element	Description of element shaping space	Key factors influencing health equity policy space
Contextual factors	The overall environment (national and international/global) in which actors are engaging; “key events” that offer opportunities for change.	– Historical factors that shape overall context for engagement and actions on health equity – International/global factors that shape the opportunities for addressing health equity – Political context that shapes degree of prioritisation of health equity policy – Social and economic factors shaping opportunities for health equity policy – “Windows of opportunity” that provide moments for change for health equity policy – Range of actor views on health equity and underlying justice frames
Actor engagement	The factors shaping how actors engage with and influence policy space	– Range of actors present and engaged on issue of health equity – Level of actor agency for health equity – Actor power and use of power in policy process for health equity – Practical actions to address health equity
Policy circumstances	The nature of engagement and decision making in the policy process	– Prioritisation of health equity in policy process – Level of political urgency to address heath equity – Availability of discussion/decision-making spaces to discuss/address health equity specifically
Policy characteristics	The acceptability of the issue of health equity	– Cohesiveness of understanding/meaning of health equity – Degree of visibility of health inequity/evidence of size of health inequalities – Impact of addressing health equity on different populations – Levels of resources needed to address health equity – Identified programmes to address health equity – Links with wider political processes
Policy spaces	The nature and use of spaces to advance health equity	– Types of spaces – Use of spaces – Governance of spaces

The Myanmar case study suggests that the elements and factors in our framework are useful for examining a pre-transition period to help support a platform for action once the transition is underway. As such, this framework could also be used beyond its initial policy analysis role to support strategy development in other contexts. Friedman (2015); Friedman (2019) has called for development of health equity strategies at country level. Use of our framework could provide a useful starting point in this process through identification of current space to address health equity and further expansion of the space to help ensure strategies succeed.

The Myanmar case study emphasizes potential challenges for policymakers in addressing long-standing and deep-seated inequalities. Political, administrative and capacity issues preclude easy or rapid approaches to addressing these challenges. A particular challenge is to consider the meaning of a health equity goal (Lane *et al*. 2017). As our findings showed, not all actors gave the same meaning to health equity and not everyone was included in the definition. Coleman and Lawson-Remer argue that in developing policies to distribute health resources, all population groups should feel the benefits of transition. This poses a challenge for policymakers in Myanmar as they weigh up the advantages and disadvantages of different policy options, population perceptions and resources. The move by the military to regain full control in Myanmar in 2021 (Price, 2021) abruptly halted the country’s democratic trajectory with implications for the health equity policy space, including international responses to military action, the availability and use of international assistance (USAID, 2021), and recent analysis of a weakened economy (World Bank, 2022). Learning from the 2006 to 2016 transition described here demonstrates the need to focus on critical factors to maintain space for health equity, including discussion of equity, the availability of data for visibility of the issue and the implementation of programmes that support the most vulnerable.

The Myanmar context is unique but shares some important characteristics with other contexts. For instance, various countries are undergoing transitions, including political–economy and sociopolitical elements. Our findings resonate with challenges faced in other transitional contexts, such as Brazil, Mexico, Chile, South Africa (McIntyre and Gilson, 2002) and Cambodia (Ir *et al.*, 2010), in which political transitions presented opportunities to further pro-equity policies. However, these examples show that equity gains following transition often fall short of original intentions. This may be due to powerful groups undermining the impact of reforms and illustrates the political nature of processes to engender health equity. An appreciation of potential ‘*arenas of conflict’* identified by Grindle and Thomas (1991, p. 185) is crucial to developing effective policy and implementation strategies. Evidence from other countries also emphasizes the importance and challenges of an inclusive and ‘fair process’ for discussing how resources are shared within the country (McIntyre and Gilson, 2002) and the need for explicit discussion on equity in the policy process and clarity on the equity goal being sought (McIntyre and Gilson, 2002; Ridde, 2008).

The extent to which discussion on UHC in Myanmar and other contexts can be opened to promote wider debate on longer-term health equity is unknown. This framework provides an opportunity to explore emerging spaces within a transitioning context. At the same time, it is clear that Myanmar’s transition has specific characteristics, has been halted since 2021, and remains unrealized. Other transitions can provide additional exploration and testing of our framework.

We argue that to achieve specific policy outcomes—in this case, advancing the health equity agenda—policy space must be strengthened and used. Although policy space opened in Myanmar, particularly after 2015, certain conditions hampered the most effective use of this space to address health equity. For example, an historical ‘command and control’ approach within MoH limited the range of experience staff could draw upon to make decisions once the system changed, affecting how staff sought and used available opportunities. In addition, historically low health funding levels in Myanmar mean it will take time to ensure sufficient allocation of finances across critical areas to achieve pro-equity outcomes. The historical cost-sharing approach to health system financing, although abandoned, left a legacy of reliance on high out-of-pocket payments, impacting equity in access and use of services. The vertical nature of health programming in Myanmar over many years also limited the adoption of a holistic approach to health equity and confined discussion to specific issues. While actors working to address specific diseases have been able to use funding provided by vertical programmes, this did not extend to the wider health equity agenda. Gopalan *et al*. (2011) noted the fragmentation of efforts to address health equity resulting from the focus on donor-funded vertical programmes, and others suggested caution in assuming that vertical initiatives necessarily improve equity.

In relation to the framework, this research found that the chosen elements within the framework, individually and collectively, were useful in eliciting observations on the policy space for health equity in Myanmar. Factors identified under each framework element resonated with participants as important aspects of policy space for health equity. However, in some cases, there was insufficient detail in responses to fully illustrate the issue, while in others the interviews provided insights into factors not included in the original framework. This study also provided a sense of the relative importance of each factor, helping to present a hierarchy of factors for any future framework. For example, it confirmed the importance of context as a key element of policy space and the comparative importance of particular factors such as the ‘policy windows’ provided by successive elections in opening up spaces to move a pro-equity agenda forward. Additionally, while not an intended framework aim, use of framework also helped to identify factors that could assist development of policy space for health equity. This included elements such as data collection to aid visibility of inequalities; fostering opportunities to discuss health equity or inequalities; supporting the development of capacity to engage in policymaking within the context, and developing an understanding of health equity and its significance, at the earliest opportunity. These elements would provide appropriate inputs in any period of transition.

Several limitations should be considered when assessing whether this policy space framework is a useful tool for policy analysis. First, the small sample size and possible sensitivities about discussing equity issues may have prevented the framework being fully tested. Second, the first author was known to many interviewees, which may have resulted in some response bias. Third, some interview responses lacked depth, highlighting the need to revisit how equity is studied in future. In particular, future research should examine the impact of addressing health equity on different population groups.

## Conclusions

Given the global drive towards embedding equity in health investments and the added visibility given to health equity by the COVID-19 pandemic, conceptualizations that inform research and policy development are critical. This research developed a conceptual framework for health equity policy space that can be used to identify opportunities to promote a pro-equity approach to policy and programming, particularly in countries undergoing transition.

## Reflexivity statement

FC and NH have lived and worked in Myanmar, and TTS, TSH and WYKK are Myanmar nationals, thus most authors had worked within the Myanmar health system and were familiar with some of the interviewees. We therefore adopted an ongoing self-reflexive approach, to mitigate against different types of researcher bias. This included use of a reflective diary by the lead author and specific coding to highlight and examine interactions in interviews to help anticipate and mitigate concerns. TTS and TSH are experienced government officials, while FC, WYKK, NH and DB have postgraduate training in qualitative social science research methods. NH and DB are experienced public health researchers and supervised FC’s doctoral thesis research, of which this is a component.
